# Research on the relation of EEG signal chaos characteristics with high-level intelligence activity of human brain

**DOI:** 10.1186/1753-4631-4-2

**Published:** 2010-04-27

**Authors:** Xingyuan Wang, Juan Meng, Guilin Tan, Lixian Zou

**Affiliations:** 1 Faculty of Electronic Information and Electrical Engineering, Dalian University of Technology, Dalian 116024, China; 2 School of Information Engineering, Dalian Fisheries University, Dalian 116024, China

## Abstract

Using phase space reconstruct technique from one-dimensional and multi-dimensional time series and the quantitative criterion rule of system chaos, and combining the neural network; analyses, computations and sort are conducted on electroencephalogram (EEG) signals of five kinds of human consciousness activities (relaxation, mental arithmetic of multiplication, mental composition of a letter, visualizing a 3-dimensional object being revolved about an axis, and visualizing numbers being written or erased on a blackboard). Through comparative studies on the determinacy, the phase graph, the power spectra, the approximate entropy, the correlation dimension and the Lyapunov exponent of EEG signals of 5 kinds of consciousness activities, the following conclusions are shown: (1) The statistic results of the deterministic computation indicate that chaos characteristic may lie in human consciousness activities, and central tendency measure (CTM) is consistent with phase graph, so it can be used as a division way of EEG attractor. (2) The analyses of power spectra show that ideology of single subject is almost identical but the frequency channels of different consciousness activities have slight difference. (3) The approximate entropy between different subjects exist discrepancy. Under the same conditions, the larger the approximate entropy of subject is, the better the subject's innovation is. (4) The results of the correlation dimension and the Lyapunov exponent indicate that activities of human brain exist in attractors with fractional dimensions. (5) Nonlinear quantitative criterion rule, which unites the neural network, can classify different kinds of consciousness activities well. In this paper, the results of classification indicate that the consciousness activity of arithmetic has better differentiation degree than that of abstract.

## Introduction

EEG signal is a spontaneous bioelectricity activity that is produced by the central nervous system. It includes abundant information about the state and change of the neural system; therefore it is widely used in clinic and neural-electricity physiological research. In recent years, with the development of the nonlinear dynamics, more and more evidences indicate that the brain is a nonlinear dynamic system, and EEG signal can be regarded as its output [1,2]. In 1985, Babloyantz *et al*. first put forward that II and IV stage EEG signals of human sleep cycle are chaotic [3]. Hereafter, a large number of study results were reported that the EEG was derived from chaotic systems [4-8]. Therefore, people try to analyze EEG signals by way of nonlinear dynamics to get new knowledge of the brain. Lindenberg, Lehnertz and Ferri *et al*. researched several kinds of physiological and pathologic conditions; and computed the relevant data under various conditions. They point out finally, the nonlinear characteristic of the physiological EEG signals greatly differs from that of the pathology; when clear-headed, the brain has higher chaotic degree, processes information more quickly and can make more responses [9-12]. Chaos is unordered, but in some situations, it has organizing structures and high order and is the source of system information [13]. Therefore, in this paper, we study the relation of chaos characteristic of EEG signals with high-level intelligence activity of human brain through comparative studies of the nonlinear dynamic characteristic of the dynamic physiological EEG information of brain under different consciousness conditions.

### Theory and Method

Chaotic system is described by strange attractors in the phase space [13]. In order to construct the phase space, we adopt the phase space reconstruct technique which was put forward by Packard *et al*. [14] and made reliable mathematical base by Takens [15]. Its principle is: Reconstruct *m*-dimensional phase space from EEG time series {*x*_*n *_| *n *= 1, 2,⋯, *N*}, then we get a group of phase space vectors.

where *τ *is the time-delay; *m *≥ 2*δ *+ 1, *δ *is the number of the system independent variables. *M *is less than *N *and they have the same order of magnitude. To reconstruct phase space, it is critical to analyze the phase graph, compute correlation dimension and Lyapunov exponent.

### CTM Algorithm and the Determinism Computation of EEG Signals

Whether the brain is a deterministic system, determines the applicability of the nonlinear dynamic method of studying EEG signal [16]. Generally, the deterministic computation of the EEG signal requires much data; and supposes the spread of adjacent lines of EEG series in the phase space are similar. However, unstable data often generates false results. CTM algorithm is a method to express the second-order difference plot (SODP) characteristic of trajectory tangent vector quantificationally. It can be used in the deterministic computation of nonlinear time series effectively. This algorithm is real-time, stable and anti-noisy [17]. The tangent vector of trajectory in the reconstructing phase space is

The angle between the tangent vectors can be expressed by its cosine value

Compared with the angle itself, the cosine value can resist noises better. The SODP of signal expresses the change rate of the tangent vectors angle *A*(*n *+ 2) - *A*(*n *+ 1) to *A*(*n *+ 1) - *A*(*n*), its CTM value is

The value of CTM reflects the smooth degree of the attractors' trajectory: the smaller the CTM value is, the less the changes of tangent vector angle, the smoother the trajectory is; and vice versa. The determinacy of the signal S can be measured by the ratio of the CTM value of the EEG series data and the surrogate data. The bigger S is, the stronger the randomicity of EEG signal is. The researches show: the deterministic signal S < 0.3; the random signal S > 0.7; as to part deterministic signal 0.3 < S< 0.7.

### Approximate Entropy

In 1991, Pincus put forward a rule to measure the complexity and the statistic quantification of time series, i.e., approximate entropy [18]. The approximate entropy can weigh the probability of creating new pattern of time series. The bigger the probability is, the more complex the time series gets. Because only less data is needed to compute the stable estimated value of the approximate entropy, the approximate entropy is suitable for the classification of nonsteady consciousness EEG signal. For example, the sampling frequency for most EEG machines are between 100-1000 Hz, but computing the approximate entropy needs 100-1000 data points, so the EEG data length used for classification can be taken as 0.5-1 s. Although there are false mark disturbance and power frequency disturbance while gathering EEG signals, the EEG data needed is very short. So the approximate entropy has strong anti-chirp and antijamming ability. At present, there is still dispute on whether EEG is derived from chaotic systems or disorderly linear random systems [19]. The approximate entropy is suitable for deterministic and random signal, which further shows that the approximate entropy has better practicability.

The concrete algorithm for approximate entropy is described as follows: Suppose the initial data as *x*(1), *x*(2),⋯, *x*(*N*).

(1) Form a group of *m*-dimensional vector according to the serial number order: ***X***(*i*) = [*x*(*i*), *x*(*i *+ 1),⋯, *x*(*i *+ *m*-1)] (*i *= 1, 2,⋯, *N*-*m *+ 1).

(2) Define the distance between ***X***(*i*) and ***X***(*j*) as

and compute the distance *d *[***X***(*i*), ***X***(*j*)] between ***X***(*i*) and other vectors ***X***(*j*) (*j *= 1, 2,⋯, *N *- *m *+ 1; *j *≠ *i*) for every *i *value.

(3) Given the threshold value *r*, count the number of *d*[***X***(*i*), ***X*(***j*)] which is smaller than *r *for every *i *value, and compute the ratio of this number to the total distance *N *- *m*:

(4) The average value of *i *is computed according to logarithm of :

(5) Add the dimension by 1 again to *m *+ 1, repeat steps (1) to (4), and compute  and *ϕ*^*m *^(*r*).

(6) The theoretical value of the approximate entropy is

Generally speaking, the boundary value mentioned above exists by probability 1. *N *can't be ∞ in practice. When *N *is a finite value, the result is the estimated value of *APEn *when the series length is *N*, which is defined as *APEn*(*m, r, N*) = *ϕ*^*m *^(*r*) - *ϕ*^*m*+1 ^(*r*). Obviously, the value of *APEn *is related with the value of *m *and *r*. According to Pincus's work, *m *= 2 and *r *= 0.1: 0.25*SD*_*x *_are suggested (*SD*_*x *_is the standard deviation (SD) of initial data *x*(*i*) (*i *= 1, 2,..., *N*)).

### Multi-lead Correlation Dimension

In the study of nonlinear dynamics of EEG signals, the Takens's time delay reconstruction phase space method used EEG data of single channel record to reconstruct multi-dimensional EEG attractor, which reflects the time correlation of the system. In order to show the characteristic of the system from time and space, Eckmann and Ruelle proposed the multichannel reconstructing (multivariable embedding) method that can show the correlation of space and time simultaneously. When applied in time series with short-time noise, it can avoid problems such as the choice of delayed parameters and system errors with higher embedding dimension. Rombouts *et al*. thought the multichannel reconstructing method can provide more reliable results [20]. Take EEG signals as an example, recording variable of each lead is taken as a component of the reconstructing vector while reconstructing, the reconstructing dimension is decided by the electrode number of EEG signals.

Based on the multi-lead data, the principal step of computing the correlation dimension with GP algorithm [21] is: The *m*-dimensional embedding-space {***X***} is got from *m*-lead observing time series.

(1) Suppose ***X***(*n*) = {*x*_1_(*n*), *x*_2_(n),..., *x*_*m *_(*n*)} (n ≤ N, *m ≤ M*), here *m *is the number of the required variables.

(2) For a given distance *r*, compute the correlation integral

here ***X ***is the vector in embedded space, *N *is the number of the vector, *w *is Theiler window, *H *is Heaviside function.

(3) For an enough small *r*, the correlation integral approaches to the following formula:

(4) Evaluate the slope of the fitting straight line in the linearity range of *LnC*_*m*_(*r*)~*Ln*(*r*), namely the estimated value of the correlation dimension *D*_2_.

Generally speaking, the correlation dimension of EEG represents the invariable measure for the self-similarity and the criterion irrelevance of the EEG signal, and shows the complex degree of the EEG signal.

### Small Data Sets Method of Computing Lyapunov Exponent

The ordinary method of studying whether the actual observable series has chaotic characteristic or not, is to compute the biggest Lyapunov exponent *λ*_1 _of the observable series. When *λ*_1 _> 0, the observable system is believed to be chaotic. Since Wolf proposed and computed the Lyapunov exponent according to the observable series in 1985, there are some sophisticated methods in this respect, such as Jacobian method, *p *norm method and the small data sets method proposed by Rosenstein *et al*. [22]. The small data sets method is more robust than other methods to embedded dimension of the phase space, the reconstruction time delay, observable noises and so on.

Mark the constructed phase space as ***X ***= [***X***_1_, ***X***_2_,⋯, ***X***_*N*_]^T^, phase point is ***X***_*j *_= [*x*_*j*-(*m*-1)*J*_, *x*_*j*-(*m*-2)*J*_,⋯, *x*_*j*_] (*j *= 1, 2,⋯, *N*), here *N *is the total number of the phase points, *m *is the embedding dimension of the phase space, *J *is the reconstructing time delay. Generally, *J *= *k*Δ*t*, *k *is a positive integer, Δ*t *is sampling interval. For ∀***X***_*j *_∈ ***X***, define , and , *p *is the average cycle of the time track. If ∃***X***_*j *+ *i *_∈ ***X ***and , define , then the advanced distance *d*_*j*_(*i*) has the following approximate relation(1)

here Δ*t *is the sampling interval or the step length of the observable series; *i *is the sliding step ordinal of the phase point along the time track. Take natural logarithm to both sides of the formula (1), we can get

When  (||·|| denotes the vector 2 norm), we get the empirical formula which Rosenstein *et al*. used to compute *λ*_1 _[22]. In view of the influence of local computation, the last empirical formula is

Here <·> is to get average.

### Power Spectra

Using Auto-Regressive (AR) parameter model method to compute the self power spectra estimated value of the EEG signal [23]: The AR model of the EEG time series *x*_*n *_is provided by the following formula(2)

here *p *is the order of the AR model; *a*_*k *_(*k *= 1, 2, ⋯ *p*) is AR model parameter; *w*_*n *_is the unpredictable part of *x*_*n*_, namely residual error. If the model can well match the EEG time series, *w*_*n *_should be white noise process. According to the AR model given by formula (2), we can get the estimated value of the AR spectra(3)

here  is the variance of AR model residual error. From the formulas (2) and (3), we know the key to get the AR spectra estimation is to estimate the AR parameters *a*_*k *_(*k *= 1, 2, ⋯ *p*) through the EEG time series. Usually, Yule-Walker equation and Levinson-Durbin algorithm are used to estimate AR parameters. In this paper, we use Burg algorithm. Burg algorithm is an autoregression power spectra estimated method, on the premise of Levinson-Durbin recursion restraint, making the sum of the front and back forecast error energy smallest. Burg algorithm avoids the computation of self-correlation function. It can distinguish the extremely close sine signal in low noise signals, and may use less data record to estimate, and the result is extremely close to real values. Moreover, the forecasting error filter obtaining from Burg algorithm is minimum phase.

The choice of the model order *p *is a critical problem in the AR model spectra estimate. If *p *is too low, it will cause smooth spectra estimate; while if *p *is too high, it will cause spectral line excursion and spectral line abruption and generate general statistic instability. In this paper, we adopt Akaike information criterion (AIC) to estimate the value of the order

here *N *is the number of the data points,  is the estimated value of the white noise variance (forecasting error power) of *p *order AR model.

### SOM Neural Network

The neural network is a highly nonlinear system; and it also shares similar characteristics with brain, so it is used in various classifications extensively. SOM neural network is composed of entire connection neuron array and it is a non-teacher, self-organizing and self-learning network. Its idea is that neurons in different areas of the space have different functions. When the neural network accepts an external input mode, it will be divided into different response areas, and each area has different response characteristics to the input mode.

A typical characteristic of SOM network is that it can generate the characteristic topology classification of input signal on one-dimensional or two-dimensional processing unit array, so the SOM network can extract the pattern characteristics of the input signal. Generally, SOM network only includes one-dimensional array and two-dimensional array, but it can also be generalized into multidimensional processing unit array. This research uses two-dimensional array. SOM network is made up of the following four parts.

(1) Processing unit array. Using to accept the input event and forming "discriminant function" of these signals.

(2) Comparison and choice of mechanism. Using to compare the "discriminant function". And choosing one processing unit which has the biggest output value.

(3) Partial interconnection action. Using to drive the chosen processing unit and the processing unit closest to it simultaneously.

(4) Adaptive process. Using to revise the parameter of driven unit in order to increase its output value to the specific input "discriminant function".

## Experiment and Result

### EEG Data Source

**Figure 1:**
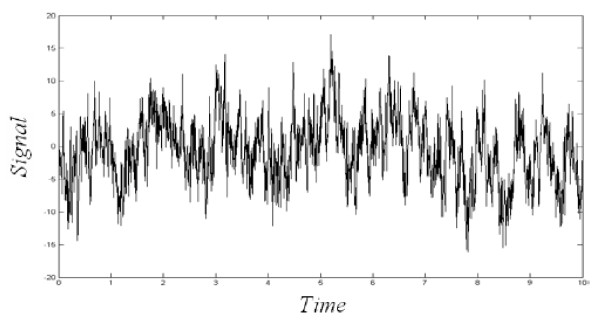
**EEG signal waveform of subject 1 while relaxing**.

The data used in this paper is the consciousness activities EEG data of 7 subjects that offered by the EEG research center of Colorado State University [24] There are five kinds of human consciousness activities, i.e. relaxation, mental arithmetic of multiplication, mental composition of a letter, visualizing a 3-dimensional object being revolved about an axis, and visualizing numbers being written or erased on a blackboard [25]. The experimental process of data acquisition is: Subjects sit in the sound-insulated and light-weak room with the electrode cap and complete some consciousness tasks according to the indications. The corresponding electrical signals of the brain will be recorded. The electrode is laid in C3, C4, P3, P4, O1, O2 and EOG(Electro-Oculogram) altogether 7 leads according to international 10~20 system standard. The sampling frequency is 250 Hz, the simulative filtering range is 0.1~100 Hz. Signals polluted seriously by winks are excluded. Experimental data of each consciousness task last 10 s. Fig. 1 is the EEG signal of subject 1 while relaxing. It is obvious that even under relaxing conditions; healthy people's EEG signals fluctuate in a complicated way, which contains abundant nonlinear dynamic information.

### Phase Graph Analysis

**Figure 2:**
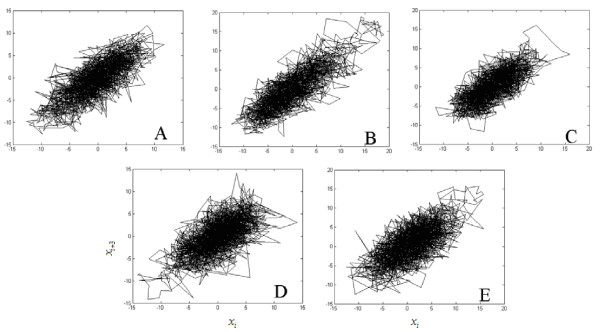
**EEG attractors of five kinds of consciousness activities of subject 1**. (a) Relaxation. (b) Mental arithmetic of multiplication. (c) Mental composition of a letter. (d) Visualizing a 3-dimensional object being revolved about an axis. (e) Visualizing numbers being written or erased on a blackboard.

Using the phase space reconstruct technique from one-dimensional time series to determine the time delay *τ *: In the experimental system, it should be through repeated trial method to confirm choice of *τ*. If *τ *is undersize, the track of the phase space will approach to a straight line; per contra *τ *is oversize, the data point will centralize in a small range of the phase space, and we can't get the attractors' local structures from the reconstructed phase graph [13]. Testing repeatedly, we find that selecting *τ *= 3, data point *N *= 2000, it can well reconstruct the EEG attractors. We construct the EEG attractors of all five kinds of consciousness activities of 7 subjects and find that EEG attractors of various patterns have similar characteristics. Fig. 2 is a representative one. As can be seen from Fig. 2, the attractors' track often rotate in an extremely complex way, even smear a group black in the plane, but there is still internal structure when the attractors is magnified. The attractors of relaxation, mental composition of a letter and visualizing a 3-dimensional object being revolved about an axis often distribute in a small ellipse region, while the point in the attractors of mental arithmetic of multiplication and visualizing numbers being written or erased on a blackboard centralize nearby the 45 degree line and there is a large distributing range along the 45 degree line. This is because while proceeding rational computation such as mathematics or imagination, the value of the adjacent sampling points of EEG signals are close, and the amplitude values of the whole EEG signals are great.

### Power Spectra Analysis

**Figure 3:**
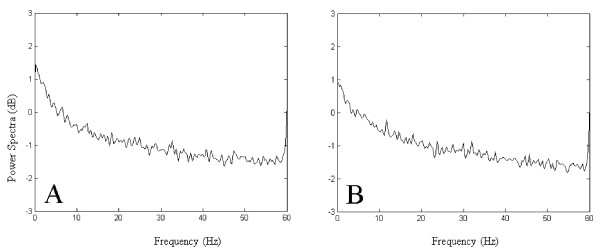
**EEG power spectra of 2 kinds of tasks of subject 1**. (a) Mental arithmetic of multiplication. (b) Visualizing a 3-dimensional object being revolved about an axis.

Using the AR parameter model method, we select 250 Hz sampling frequency to compute the power spectra of five kinds of tasks' EEG signal of 7 subjects. The parameters used in analysis are: the length of FFT *M*: 1024; the total number of the data *N*: 6000; order *p*: 320. By comparison of the power spectra of five kinds of tasks of 7 subjects, we find that the power spectra of five kinds of tasks for identical subject are similar and meet 1/*f *distribution. As can be seen from Fig. 3, although the attractors' difference is great (Fig. 2(b) and 2(d)), their power spectra (Fig. 3(a) and 3(b)) show certain similarity. The peak in the high-frequency in Fig. 3 is caused by the power frequency disturbance.

Practice prove: The EEG of human can be divided into four frequency sections: δ wave: the frequency is 1-4 Hz, appears while sleeping, anaesthetizing deeply, oxygen deficit or the brain with organic disease; θ wave: the frequency is 4-8 Hz, appears while feeling sleepy; α wave: the frequency is 8-13 Hz, appears while closing eyes with clear-headed; β wave: the frequency is 14-30 Hz, appears while opening eyes and looking at things or thinking. As can be seen from Fig. 3, although the spectral lines are similar, there are differences in the active frequency bands (8-30 Hz) of different consciousness. So we add the energy of 8-13 Hz and 14-30 Hz separately in order to use it in SOM network to classify the consciousness.

### CTM and the Deterministic Computation of the Signals

**Figure 4:**
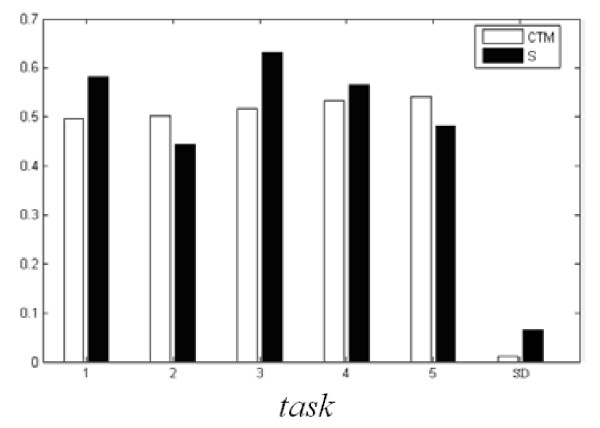
**Statistic average histogram of CTM and S of 5 tasks of subject 1 (SD denotes standard deviation)**.

EOG signal is the main disturbance of each lead EEG signal, so we make a relevant analysis separately between the gathered EOG signal and another 6 leads in order to find several leads which are disturbed less. We choose *τ *= 3 and *m *= 16 to compute the CTM. The method of surrogate data [26,27] is used to help detect nonlinear determinism. The surrogate data are linear stochastic time series that have the same power spectra as the EEG signal series. In this paper, we use "iteratively refined surrogate data", which have the same autocorrelation function, Fourier power spectrum, and probability distribution as the EEG time series. More detailed algorithms used in this study are present in the paper of Schreiber and Schmitz [27]. Fig. 4 gives the statistic average histogram for each task of 100 times testing. It is obvious that the value of CTM accords with the phase graph 3 well. The statistic average results of the deterministic computations of the EEG signals are in the interval of 0.3 < S <0.7. It offers strong support that human brain which contains chaotic component is a highly nonlinear system. But while proceeding deterministic tests, we also find that its value's fluctuation is very big. As an empirical algorithm, when there is less data sample, its application also has certain limitation.

### Approximate Entropy Computation

**Figure 5:**
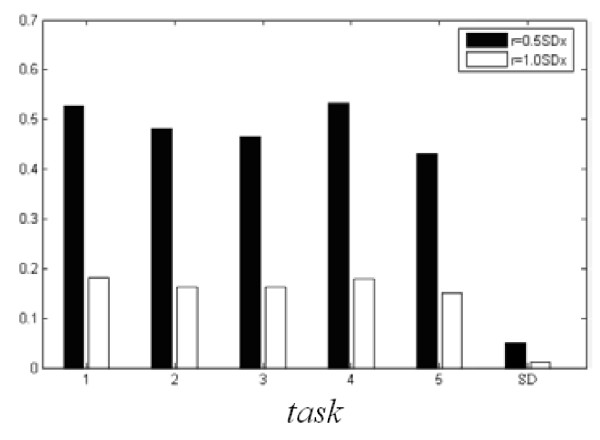
**Statistic average histogram of the approximate entropy of subject 1 when r = 0.5SDx and r = 1.0SDx**.

According to the characteristics of the processing data, we choose *r *= 0.5*SD*_*x *_and *r *= *SD*_*x*_. The approximate entropy to 100 groups of data is computed separately. Because the data gathered from different electrodes may be asynchronous, we make interval eliminations to those unsuitable data. Fig. 5 provides the statistic average histogram of the approximate entropy when *r *= 0.5*SD*_*x *_and *r *= *SD*_*x*_. From Fig. 5, we can see, the consciousness activities (task 2 and 5), with more rational consciousness such as arithmetic, have relatively weaker ability to generate new pattern; while those consciousness activities (task 4), with more abstract consciousness such as visualizing graph rotating, have relatively stronger ability to generate new pattern, which means that the time series have more complexity. This also corresponds to the practice. Because mathematical computation is based on fixed rule, its ability to create new pattern ingredient is naturally lower.

### Correlation Dimension Computation

**Figure 6:**
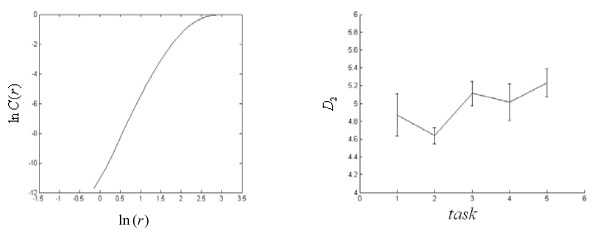
**The result of the correlation dimension *D*_2 _of subject 1**. (a) ln*C*(*r*)~ln_*r *_curve while relaxing. (b) *D*_2 _of 5 kinds of tasks and each contains10 groups of EEG data.

According to the characteristics of the processing data, we precondition the EEG data first. Namely make a relevant analysis between the EOG and other leads, and sort them according to the order from weak to strong. Then carry through the phase space reconstruction. According to the discussion by Brandstater and Swinney[13]: The fluctuation of partial derivative in scale-free region should be less than 1%. Thus, the scale-free region can be determined. Then the least square method can be used to obtain the correlation dimension. After iterative trials, we found that the correlation dimension can be exactly determined with *m *> 12. Therefore, in this experiments, we choose *τ *= 3 and *m *= 16 to compute the data of 4 subjects and each contains ten groups separately. Fig. 6(a) is a representative curve ln*C*(*r*)~ln*r *of subject 1 while relaxing. Fig. 6(b) provides the statistic results of the correlation dimension *D*_2 _of 10 groups of data of five kinds of human consciousness activities (each vertical line represents the mean square error range of each task, the crossing point between the crosswise fold line and the vertical line is the mathematic expectation of the task). We can see from Fig. 6(b): For the same subject, do the same kind of tests in different time, its *D*_2 _value may have great fluctuation, which means human brain has different excitable degree in different time slice. Fig. 6(b) also shows that the error fluctuation of *D*_2 _is minimum when implementing mathematical computation (task 2). This is because mathematical computation can make the spirit centralized more easily than other consciousness activities. In addition, we also compute the data of *D*_2 _for other 3 subjects and each contains 10 groups of data. These *D*_2 _will also be used in the ideology classification of the SOM.

### Lyapunov Exponent Computation

**Figure 7:**
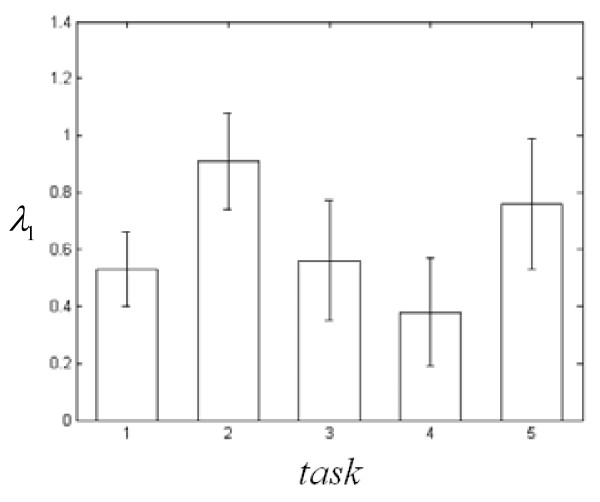
***λ*_1 _of 5 kinds of tasks and 10 groups of EEG data of subject1**.

Different consciousness activities stimulate different cerebrum regions, so the computation of single lead signal can't reflect the synthetic Lyapunov exponent of the brain consciousness activity well. The embedded dimension *m *is determined by iteratively trials. For the delay *τ*, the phase space of EEG signals is projected into the two-dimension plane. If *τ *is too small, the attractors will muster around the line *y *= *x*. If *τ *is too large, *m*·*τ *will be much more than the average period. On this basis, *τ *is determined by iteratively trials. Furthermore, considering the fact that for different consciousness, different cerebrum region has different activity degree, implement sample splicing to the sampling data of each lead with *τ *= 3, *m *= 16 to reconstruct the phase space. Fig. 7 is the biggest Lyapunov exponent *λ*_1 _of 10 groups of EEG data with five kinds of human consciousness activities (each vertical line represents the result of mean square error range of each task, the crossing point between the histogram and the vertical line is the mathematic expectation of the task). It is obvious that the biggest Lyapunov exponents *λ*_1 _of five human consciousness activities are all bigger than zero, which proves that human brain activity is chaotic.

### SOM Network Consciousness Classification

**Figure 8:**
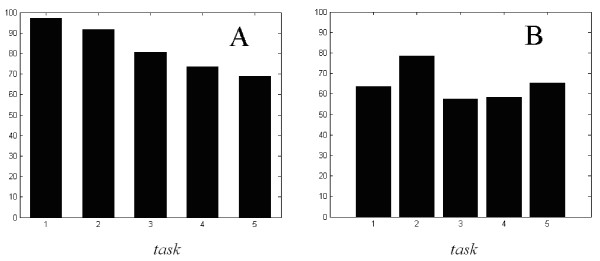
**The predicted result of SOM network**. (a) the correct resolution histogram of mixed tasks of single subject. (b) the correct resolution histogram of 4 subjects and 5 kinds of tasks'.

The purpose of the investigation in this paper is to classify the intelligence consciousness activities. From the analysis above, we know that for the same subject, the methods described above may have better differentiation degree; but for different subjects, the above methods have difficulties to classify the consciousness activities, which also indicates that the brain is a highly complicated nonlinear system. Therefore, we make the nonlinear criterions (mentioned above) into the prophase processing module, and input them to the input unit of the SOM network. That is, the SOM network has six inputs, including power spectra, CTM, S, approximate entropy, correlation dimension and Lyapunov exponents. According to the tests of the data, the competitive layer of the network is chosen as 8 × 6 structure. The predicted results of the network are shown in Fig. 8. In Fig. 8, the horizontal ordinate denotes the five outputs of the SOM network, and the vertical ordinate denotes the correct resolution. Fig. 8(a) shows the correct resolution histogram of mixed tasks of single subject. In Fig. 8(a), the outputs of the SOM network are the mixed tasks which are combined in turn from the five kinds of human consciousness activities, i.e. relaxation, mental arithmetic of multiplication, mental composition of a letter, visualizing a 3-dimensional object being revolved about an axis, and visualizing numbers being written or erased on a blackboard. For example, "3" represents the combination of three tasks, i.e. relaxation, mental arithmetic of multiplication, and mental composition of a letter. Fig. 8(b) shows the correct resolution histogram of four subjects. In Fig. 8(b), the outputs of the SOM network are the five individual tasks mentioned above. As can be seen from Fig. 8(b), the resolution of mathematical computation is relatively higher, while the resolutions of other tasks are about equivalent. The authors think that this is because the nonlinear quantitative parameters of the mathematical computation have great difference compared with other tasks. As can be seen from Fig. 8(a) and Fig. 8(b), the resolution of multi-individual drops obviously relative to single individual. This is because the nonlinear quantitative parameters of two subjects differ greatly, which makes the resolution of the network details drop. There will be better results if there are more individuals to train the network.

## Discussion and Conclusion

(1) In this paper, we use the determinacy, the phase graph, the power spectra, the approximate entropy, the correlation dimension and the Lyapunov exponent method etc to study the EEG signal of 5 kinds of consciousness activities of 7 subjects. Although every method has merits and faults, the results show the nonlinear dynamic characteristics of the subject's brain from different perspective. Thereinto, from the deterministic computation we know that the EEG signal is between random signal and deterministic signal. This indicates that the brain may be a chaotic system. The analysis of the power spectra shows that various ideology of single subject is almost identical, but the activity frequency channels for different consciousness activities are different slightly. The analysis of the approximate entropy presents the degree of various consciousness activities on generating new pattern. The approximate entropy of different subjects exist discrepancy. The authors think that at the same state, the larger approximate entropy of the subject, the more innovational he has. The correlation dimension shows the change of chaos of different consciousness activities well, which can better indicate the activity degree of human consciousness, combining with the approximate entropy and the Lyapunov exponent. The above analyses indicate: Different consciousness activities have profound nonlinear dynamic differences. Some differences are difficult to perceive, and the nonlinear quantitative parameters of different individuals have great differences. So it is a critical problem to find a widely applicable criterion, which needs to be explored for a long time.

(2) By analyzing the EEG signal of 5 kinds of human consciousness activities, the authors classify the EEG signal through SOM network. The result is almost satisfying. Because the neural network used in this research is classical SOM network, its self-applicability is rather bad. If it can be improved and applied to more samples, there will be better results.

(3) The study on profound intelligence activity of human brain needs to integrate the achievements in the fields of life science, physics and modern mathematics. It needs multi-disciplinary cooperation in many aspects, especially the new branch in recent 20 years in mathematic-physics, i.e., the nonlinear theory, to stand on a new height to scan the intelligence activity problem of human brain to achieve the purpose of providing correct quantitative criteria for the intelligence activity of human brain. The task is very arduous. Therefore the theoretical and experimental works of this research should be furthered in the future.

## Competing interests

The authors declare that they have no competing interests.

## Authors' contributions

XW proposed the basic theory and method. JM participated in the improvement of the basic method and the revision of the manuscript. GT carried out the experiments and drafted the manuscript. LZ performed the analysis. All authors read and approved the final manuscript.
